# Rare Case of Vaccination

**Published:** 1876-02

**Authors:** 


Rare Case of Vaccination.—Dr. A. Muller reports a case (*Gazette Obstetricale*) of a child aged thirteen months, vaccinated on the 9th of July. At the time it was perfectly healthy. Two days later measles declared itself. All trace of the vaccination totally disappeared. On July 17th, eight days after the date of vaccination, when the symptoms of measles were disappearing, an inflammatory areola was discerned round the points where vaccination had been performed, and from this date the progress of the vaccination was normal.

